# Long‐term Outcomes of Catheter Ablation of Atrial Fibrillation: A Systematic Review and Meta‐analysis

**DOI:** 10.1161/JAHA.112.004549

**Published:** 2013-04-24

**Authors:** Anand N. Ganesan, Nicholas J. Shipp, Anthony G. Brooks, Pawel Kuklik, Dennis H. Lau, Han S. Lim, Thomas Sullivan, Kurt C. Roberts‐Thomson, Prashanthan Sanders

**Affiliations:** 1 Centre for Heart Rhythm Disorders (CHRD), University of Adelaide and Royal Adelaide Hospital, Adelaide, Australia (A.N.G., N.J.S., A.G.B., P.K., D.H.L., H.S.L., T.S., K.C.R.T., P.S.)

**Keywords:** ablation, arrhythmia, atrial fibrillation, long‐term outcomes

## Abstract

**Background:**

In the past decade, catheter ablation has become an established therapy for symptomatic atrial fibrillation (AF). Until very recently, few data have been available to guide the clinical community on the outcomes of AF ablation at ≥3 years of follow‐up. We aimed to systematically review the medical literature to evaluate the long‐term outcomes of AF ablation.

**Methods and Results:**

A structured electronic database search (PubMed, Embase, Web of Science, Cochrane) of the scientific literature was performed for studies describing outcomes at ≥3 years after AF ablation, with a mean follow‐up of ≥24 months after the index procedure. The following data were extracted: (1) single‐procedure success, (2) multiple‐procedure success, and (3) requirement for repeat procedures. Data were extracted from 19 studies, including 6167 patients undergoing AF ablation. Single‐procedure freedom from atrial arrhythmia at long‐term follow‐up was 53.1% (95% CI 46.2% to 60.0%) overall, 54.1% (95% CI 44.4% to 63.4%) in paroxysmal AF, and 41.8% (95% CI 25.2% to 60.5%) in nonparoxysmal AF. Substantial heterogeneity (I^2^>50%) was noted for single‐procedure outcomes. With multiple procedures, the long‐term success rate was 79.8% (95% CI 75.0% to 83.8%) overall, with significant heterogeneity (I^2^>50%).The average number of procedures per patient was 1.51 (95% CI 1.36 to 1.67).

**Conclusions:**

Catheter ablation is an effective and durable long‐term therapeutic strategy for some AF patients. Although significant heterogeneity is seen with single procedures, long‐term freedom from atrial arrhythmia can be achieved in some patients, but multiple procedures may be required.

## Introduction

Catheter ablation of atrial fibrillation (AF) has become an established therapeutic modality for the treatment of patients with symptomatic AF.^1^ To date, studies reporting outcomes of AF ablation have predominantly limited follow‐up to 1 to 2 years after the index ablation procedure.^2–4^ Although the long‐term efficacy of AF ablation is less precisely defined, it is of critical relevance to individual patient prognosis, clinical decision making, and reimbursement policies for the procedure. Until recently, few series have presented the long‐term outcomes of AF ablation at ≥3 years of follow‐up. In the current study, we systematically reviewed the medical literature to evaluate the long‐term single‐ and multiple‐procedure efficacy of AF ablation.

## Methods

The study was conducted in accordance with principles established for meta‐analyses of observational studies.^5^ We searched PubMed, Embase, Web of Science, and Cochrane Database for published articles describing long‐term outcomes in patients undergoing catheter ablation of AF. The search design was conducted with the assistance of a research librarian, and the detailed search methodology is presented in Appendix S1. This search was supplemented by hand‐searching bibliographies of published studies and relevant review articles. Citations were included if they involved an evaluation of percutaneous catheter ablation outcomes at ≥3 years after the index ablation procedure, with a mean/median follow‐up of ≥24 months. Randomized controlled trials, case–control studies, cohort studies, and case series were included. Individual case reports, editorials, review articles, and meeting abstracts were excluded. Studies published in languages other than English were excluded. Studies involving surgical AF ablation and AV nodal ablation, or exclusive right atrial ablation, were excluded. The search was conducted on July 8, 2011. Citations were appraised by 3 independent reviewers (A.G., N.J.S., A.G.B.), with differences resolved by consensus. Selected publications were analyzed for the following outcomes: (1) primary ablation success—defined as cumulative survival free of recurrent atrial arrhythmia; (2) multiple‐procedure success—defined as cumulative survival free of atrial arrhythmia, including patients receiving >1 ablation procedure; and (3) number of patients undergoing multiple procedures. We included data presented as Kaplan–Meier analyses or actuarial recurrence rates. Latest follow‐up was defined as the latest follow‐up time point with ≥30 patients at risk. The definitions of postprocedure blanking period and use of antiarrhythmic drugs were left to individual study design. If on‐drug and drug‐free success data were available, drug‐free success data were included in the statistical analysis. Study data were clarified with original investigators if required. Study quality was assessed using a modified version of quality assessment criteria for case series.^6^

### Statistical Analysis

Cumulative survival data were obtained from each study and pooled at the 1‐year follow‐up and latest follow‐up time points. For Kaplan–Meier data, arrhythmia‐free survival rates were extracted using graphic digitization software (DigitizeIt). In the absence of standard errors for each Kaplan–Meier curve, the number at risk at the time point of interest was used to conservatively estimate the standard error. A pooled estimate of survival at 12 months and the latest follow‐up was calculated, using random‐effects models based on logit transformed proportions.^7^ The time point of latest follow‐up in a study was defined as the last time point reporting a minimum of 30 subjects at risk. A minimum of 3 studies was required to perform meta‐analysis. Heterogeneity was assessed with the I^2^ statistic, with 50% defined as the threshold for significant heterogeneity.^8^ Subgroup analysis and random‐effects meta‐regression were performed to explore possible reasons for heterogeneity of study outcomes. Evidence for publication bias was assessed graphically using funnel plots. For the number of procedures per patient, exact Poisson CIs were calculated around each study estimate. Study estimates and CIs were then pooled using random‐effects models. Statistical analysis was performed with Comprehensive Meta‐Analysis software, version 2 (Biostat) and STATA version 11 (StataCorp).

## Results

### Search and Synthesis of Literature

**Figure 1.**
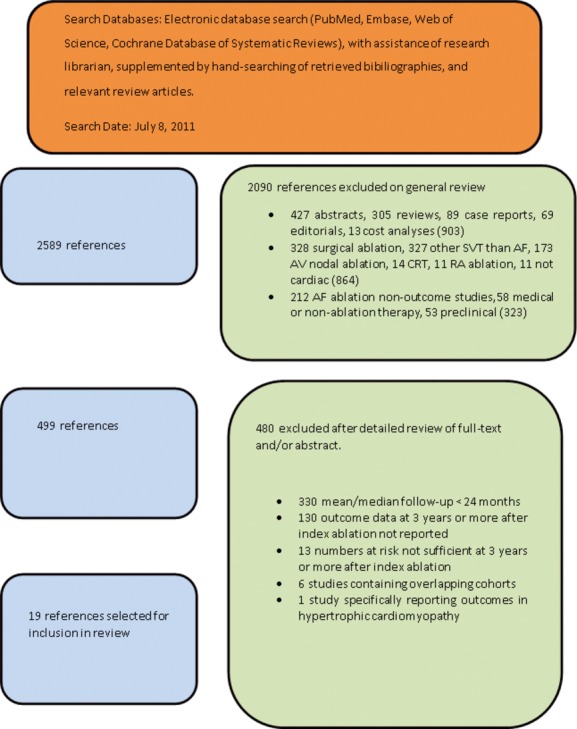
Search criteria and flow diagram for studies included in this systematic review. AF indicates atrial fibrillation; SVT, supraventricular tachycardia; AV, atrioventricular; CRT, cardiac resynchronization therapy; RA, right atrial.

We identified 2589 unique citations after the initial literature search was combined with supplementary hand searches; 2090 were excluded after screening of abstracts and titles, and 480 were selected for detailed secondary review of the full text and/or abstract. A total of 19 publications were identified that met the inclusion criteria (Figure 1).^9–27^ Baseline characteristics of these 19 studies are presented in Table 1. Included studies were published in 2003–2011, with study enrolment from 1998 to 2009. The included studies consisted of 15 single‐center case series, 2 multicenter case series, and 2 randomized controlled trials. Prospective recruitment occurred in 7 studies,^13–15,13–18,13–25^ with 12 studies recruiting retrospectively.^9–12,16,19,21–23,21–27^ Study size varied considerably, from small (Katritsis et al, N=39 patients)^13^ to much larger studies (Bhargava et al, N=1404 patients.^17^ Eleven studies reported outcome data for paroxysmal AF (PAF) patients.^11,13–18,13–22,25^ Six studies reported data for nonparoxysmal AF (NPAF) patients.^15,17–18,17–27^ Six studies provided overall outcome data for mixed PAF/NPAF cohorts.^9–10,12,19,24–25^

**Table 1.** Baseline Characteristics for Patients Included in Systematic Review

First Author, Year	Study Design	Inclusion Criteria	Comparator Intervention Groups	N	Age, y	Male, %	PAF, %	LA Diameter, mm	LVEF, %
Pappone, 2003^9^	Prospective single‐center nonrandomized case–control study	Consecutive AF patients, assigned to catheter ablation or medical therapy according to patient/clinician preference	PVAI	589	65	58	70	46	54
			Medical therapy	582					
Pratola, 2008^9^	Retrospective single center	Symptomatic drug‐refractory PAF/persistent AF	—	72	63	57	42	42	57
Sartini, 2008^9^	Retrospective single center	Symptomatic drug‐refractory PAF	—	139	55	73	100	41	67
Shah, 2008^9^	Retrospective single center	Follow‐up study of 264 patients free of AF 1 y after PVI; 350 patients initially ablated	—	264	56	65	86	38	56
Katritsis, 2008^9^	Prospective single center	Symptomatic PAF	—	39	52	87	100	40	
Fiala, 2008^9^	Prospective single‐center randomized controlled trial	Symptomatic PAF	PVI (segmental isolation)	54	51	80	100	38	59
			PVI (circumferental isolation with electroanatomic mapping)	56	53	82	100	40	
Gaita, 2008^9^	Prospective single‐center randomized controlled trial	Symptomatic PAF and persistent AF	PVI	67	53	82	61	44	
			PVI plus linear ablation	137	56	78	61	46	
Sawhney, 2009^9^	Retrospective single center	Symptomatic PAF	—	71	60	79	100	39	56
Bhargava, 2009^9^	Prospective multicenter	Symptomatic AF	—	1404	56	76	52	43	55
Bertaglia, 2010^9^	Retrospective multicenter	Follow‐up study of 177 patients free of AF 1 y after PVI; 229 patients initially ablated	—	177	59	75	58	46	58
Tzou, 2010^9^	Retrospective single center	Follow‐up study of 123 patients free of AF 1 y after PVI; 239 patients initially ablated	—	123	54	80	85	43	56
Hunter, 2010^9^	Retrospective single center	AF patients undergoing catheter ablation	—	285	57	75	53	43	—
Ouyang, 2010^9^	Retrospective single center	Symptomatic PAF		177	60	75	100	43	—
Medi, 2011^9^	Retrospective single center	Symptomatic PAF		100	54	79	100	42	59
Matsuo, 2011^9^	Retrospective single center	AF patients undergoing catheter ablation		260	54	90	59	39	66
Weerasooriya, 2011^9^	Prospective single center	Symptomatic drug‐refractory AF		100	56	86	64	—	70
Hussein, 2011^9^	Prospective single center	Symptomatic drug‐refractory AF		831	59	78	59	47	53
Rostock, 2011^9^	Retrospective single center	Persistent AF		395	60	80	0	47	59
Winkle, 2011^9^	Retrospective single center	Symptomatic AF		843	62	72	32	—	—

PAF indicates paroxysmal AF; LA, left atrial; LVEF, left ventricular ejection fraction; AF, atrial fibrillation; PVAI, pulmonary vein antral isolation; PVI, pulmonary vein isolation.

Study quality was assessed using a modified version of quality assessment criteria for case series.^6^ Study quality was generally limited, with the majority of studies having identifiable limitations in study design (Appendix S2). Patient entry criteria were generally well defined. However, important features, such as study design including consecutive recruitment, losses to follow‐up, and prognostic factors for recurrence or ablation success, were only variably reported.

### Baseline Patient Characteristics

A total of 6167 patients were included from the 19 studies. The mean age of patients in the included studies ranged from 51 to 65 years (Table 1). All studies predominantly included male subjects, with the proportion of male subjects varying from 57% to 90% (Table 1).The mean left atrial diameter varied from 38 to 47 mm and the mean left ventricular ejection fraction varied from 53% to 70% (Table 1).

### Catheter Ablation Approach

Pulmonary vein (PV) isolation using radiofrequency energy was the method of ablation in the majority of included studies (Table 2). A wide area or PV antral circumferential ablation strategy was used in 10 studies.^11,14–15,14–18,14–22,25,27^ Segmental PV isolation was used in 4 studies.^11,13–14,23^ The stepwise ablation technique was the procedure of choice in 2 studies.^24,26^ Two studies used an anatomical electroanatomic map‐guided ablation approach without PV isolation as an end point.^9–10^ One study reported outcomes after selective ablation of arrhythmogenic veins defined by ectopic firing in the baseline state or presence of isoproterenol.^19^ The 2 randomized controlled trials reported comparisons of ablation strategies.^14–15^ Early studies tended to use nonirrigated conventional ablation catheters, with later published studies using predominantly irrigated ablation catheters.

**Table 2.** Ablation Strategy and Follow‐up

First Author, Year	Enrolment Period	Ablation Strategy	Linear Ablation	Catheter Type	Mean/Median Follow‐up Duration, mo	Use of Antiarrhythmic Drugs	Follow‐up Year 1	Follow‐up After Year 1
Pappone, 2003^9^	1998–2001	Anatomic circumferential ablation	✗	Nonirrigated	28	✗	Clinic visit and Holter monitor 1, 3, 6, 9, and 12 mo and 6 mo thereafter Recurrence defined as symptomatic AF >10 min	—
Pratola, 2008^9^	2001–2004	PVI (segmental) or anatomic circumferential ablation	✗	3.5 mm irrigated	42	✗	Clinic visit and 24‐Holter monitor at 1, 3, 6, 9, and 12 mo	Clinic visit and Holter monitor at least 6‐monthly
Sartini, 2008^9^	2001–2004	PVI (segmental) 63, then WACA 76, CTI if inducible or previous atrial flutter	✗	8‐mm nonirrigated	33	✓	Clinic visit and Holter monitor at 1, 3, 6, 9, and 12 mo	Routine clinical follow‐up
Shah, 2008^9^	ND	PVI (segmental)	✓ (at redo)	4‐mm nonirrigated	28	✗	Clinic visit at 1, 3, 6, 9, and 12 mo and 24‐h Holter monitor at 3 moTranstelephonic monitoring for 3 mo	Clinic visit annually
Katritsis, 2008^9^	ND	PVI (segmental)	✗	4‐mm nonirrigated	42	✓	Clinic visit and Holter monitor at 1, 3, 6, 9, and 12 mo	Clinic visit and ECG every 3 mo Clinical assessment included fellows blinded to treatment
Fiala, 2008 (fluoro)^9^	2001–2003	PVI (segmental)	✗	4‐mm nonirrigated	48	✗	Clinic visit and Holter monitor at 6 wk and then at 3, 6, 9, and 12 mo	At least 6‐monthly clinic visit and Holter monitor
Fiala, 2008 (EAM) ^9^	2001–2003	WACA	✗	4‐mm nonirrigated	48	✗	Clinic visit and Holter monitor at 1, 3, 6, 9, and 12 mo	At least 6‐monthly clinic visit and Holter monitor
Gaita, 2008 (PVI) ^9^	ND	WACA	✗	Irrigated Navistar Thermocool	41.4	✗	Clinic visit and Holter monitor at 1, 3, 6, 9, and 12 mo	—
Gaita, 2008 (PVI+linear ablation) ^9^	ND	WACA+linear ablation	✓	Irrigated Navistar Thermocool	39.7	✗	Clinic visit and Holter monitor at 1, 3, 6, 9, and 12 mo	At least 6‐monthly clinic visit and Holter monitor
Sawhney, 2009^9^	2002–2003	PVI (segmental)	✓ (at redo)	8‐mm nonirrigated	63	✗	Clinic visit and Holter monitor at 1, 3, 6, 9, and 12 mo	At least 6‐monthly clinic visit and Holter monitor
Bhargava, 2009^9^	2001–2006	WACA (ICE‐guided)	✓ (in NPAF)	8‐mm nonirrigated or 3.5 mm irrigated	56.1	✗	Clinic visit and Holter monitor at 1, 3, 6, 9, and 12 mo	—
Bertgalia, 2010^9^	2001–2003	WACA	✗	8‐mm nonirrigated or 3.5 mm irrigated	49.7	✗	Clinic visit and Holter monitor at 1, 3, 6, 9, and 12 mo	At least 6‐monthly clinic visit and Holter monitor up to 36 mo, with ongoing clinic follow‐up
Tzou, 2010^9^	2001–2003	PVI (segmental arrhythmogenic vein ablation)	✗	4‐mm or 8‐mm nonirrigated	71	✓	Clinic visit at 6 wk, 6 mo, 1 y 4‐Week transtelephonic monitoring and 3 to 9 mo postablation	Nonmandatory annual clinic follow‐up, or research personnel contact by telephone or with referring providers
Hunter, 2010^9^	2001–2006	WACA	✓	Irrigated	40	✓	Clinic visit and Holter monitor at 3 and 6 mo Symptom‐related follow‐up after 6 mo	Symptom‐related follow up Contact with referring physician by research team (96% success)
Ouyang, 2010^9^	2003–2004	CPVI (double lasso)	✗	3.5 mm, irrigated	58	✗	Clinic visit and Holter monitor at 3, 6, 9, and 12 mo	At least 6‐monthly clinic visit and Holter monitor
Medi, 2011^9^	ND	PVAI	✗	4 mm, D‐curve, irrigated	39	✗	Clinic visit and Holter monitor at 3, 6, 9, and 12 mo	At least 6‐monthly clinic visit and Holter monitor
Matsuo, 2011^9^	ND	PVI (segmental)+CFAE in long‐standing persistent AF	✗	8 mm nonirrigated	30	✗	Clinic visit and Holter monitor at 1, 3, 6, 9, and 12 mo	At least 6‐monthly clinic visit and Holter monitor
Weerasoo‐riya, 2011^9^	2001–2002	Segmental ostial PVI + stepwise ablation	✓ (in NPAF)	5 mm, D‐curve, irrigated	60	✗	Holter monitor at 1, 3, 6, 9, and 12 mo	Rehospitalization of patients at 5 y postprocedure, with ECG and Holter monitor
Hussein, 2011^9^	2005	WACA+SVC ablation	✓	Irrigated	55	✗	Holter monitor at 3, 6, and 12 mo Event recorder at 3 mo	Yearly follow‐up recommended but not mandatory for non‐Cleveland Clinic patients AF registry scrutinized by research team
Rostock, 2011^9^	2007–2008	Stepwise PVI+electrogram‐guided ablation.	✓	3.5 mm irrigated‐tip	27	✓	Clinic visit and Holter monitor at 3, 6, 9, and 12 mo	At least 3‐monthly clinical follow‐up with Holter monitor
Winkle, 2011^9^	2003–2009	CPVI and roof line	✓	8 mm nonirrigated or 3.5 mm irrigated	29	✗	Daily transtelephonic monitoring for 3 mo Clinic visit and Holter monitor at 3 mo	6 to 12 monthly clinic contact or follow‐up with research nurse

✗ signifies that this approach not used and ✓ that this approach was used in the study. PVI indicates pulmonary vein isolation; AF, atrial fibrillation; ND, not dated; CTI, cavotricuspid isthmus; ECG, electrocardiogram; EAM, electroanatomic mapping; WACA, wide area circumferential ablation; ICE, intracardiac echocardiography; NPAF, nonparoxysmal AF; PVAI, pulmonary vein antral ablation; CPVI, circumferential pulmonary vein isolation; CFAE, complex fractionated atrial electrogram.

### Follow‐up

The mean or median duration of follow‐up in included studies varied from 28 to 71 months (Table 2). Follow‐up intensity differed between studies. The majority of studies (13/15) conducted a clinic visit with ≥24‐hour Holter monitoring and electrocardiography on ≥4 occasions in the first year after index ablation. After the first year, follow‐up intensity was generally reduced in most studies (Table 2). Although some studies continued at least 3 to 6 monthly clinic visit and Holter monitoring, other studies reported outcome results beyond 1 year based on data from referring clinicians or direct contact by research personnel with patients. One study rehospitalized patients at 5 year for inpatient Holter, electrocardiographic, and exercise stress testing.^24^ One study assessed AF recurrence based on a prospective AF registry.^25^

### Single‐Procedure Efficacy of Catheter Ablation

**Figure 2.**
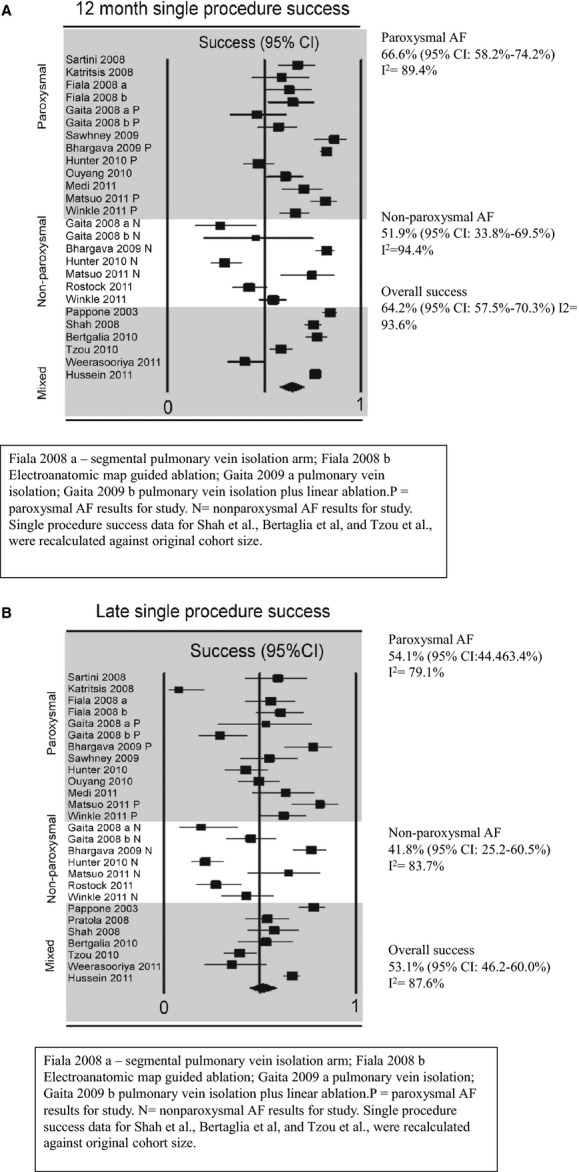
Single‐procedure success at 12 months postprocedure (A) and at late follow‐up (B). AF indicates atrial fibrillation.

Outcome data regarding the efficacy of catheter ablation of AF were available in all studies. Most studies provided single‐procedure success rates, defined as the percentage of patients free of atrial arrhythmia or not requiring a second procedure at 12 months. The pooled overall success rate was 64.2% (95% CI 57.5% to 70.3%, Figure 2A). The pooled 12‐month success rate for the 11 studies reporting outcomes for PAF patients was 66.6% (95% CI 58.2% to 74.2%, Figure 2A), and for the 6 studies reporting outcomes for NPAF patients, it was 51.9% (95% CI 33.8% to 69.5%, Figure 2A). Heterogeneity exceeded 50% in each of these groups. At late follow‐up, the overall single‐procedure success, defined as freedom from atrial arrhythmia at latest follow‐up, was 53.1% (95% CI 46.2% to 60.0%, Figure 2B). Mean long‐term success in the studies separately reporting PAF outcome was 54.1% (95% CI 44.4% to 63.4%, Figure 2B), and in the 4 studies reporting NPAF outcome, it was 41.8% (95% CI 25.2% to 60.5%, *P*=0.3 versus PAF, Figure 2B). I^2^ exceeded 50% for long‐term single‐procedure outcome data, indicating significant heterogeneity (Figure 2B). Long‐term single‐procedure outcomes for long‐standing persistent AF were reported in 3 studies,^17,23,27^ but meta‐analysis was not performed due to small numbers (<10 patients) of patients at late‐term follow‐up in 2 of these studies.^23,27^

We specifically evaluated the impact of segmental compared with circumferential PV isolation. There was no statistical difference in outcomes for segmental PV isolation (5 studies, 52.4% [95% CI 30.2% to 73.8%]) compared with wide antral circumferential PV isolation 51.6% (9 studies, 95% CI 42.7% to 60.4%, *P*=0.947).

We also specifically analyzed the impact of antiarrhythmic drugs by assessing late single‐procedure outcomes in the subgroup of 14 studies reporting drug‐free success. In this group, late single‐procedure success was 57.4% (95% CI 50.9% to 63.8%), which was similar to overall clinical outcomes.

### Impact of Multiple Procedures

**Figure 3.**
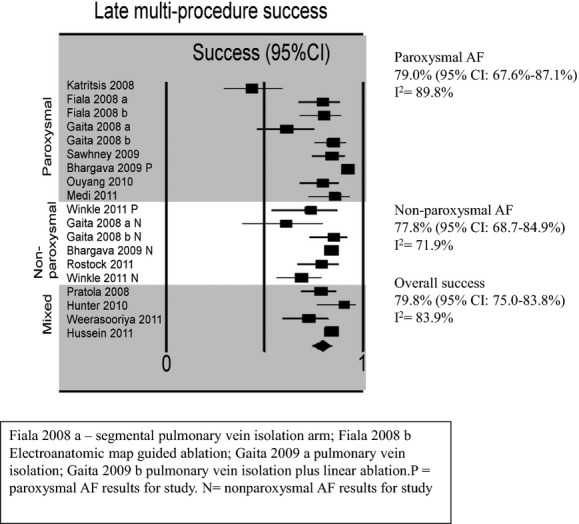
Multiple late procedure success, defined as the cumulative arrhythmia‐free survival at ≥3 years. AF indicates atrial fibrillation.

Thirteen studies provided outcome data taking into consideration the impact of multiple procedures. The overall multiple‐procedure long‐term success rate was 79.8% (95% CI 75.0% to 83.8%) in 13 studies (Figure 3). The I^2^ overall was >50%, indicating significant heterogeneity. The multiple‐procedure long‐term success in PAF was 79.0% in 8 studies (95% CI 67.6% to 87.1%, Figure 3), and that in NPAF was 77.8% in 4 studies (95% CI 68.7% to 84.9%, *P*=0.9 versus PAF, Figure 3). In the individual groups, heterogeneity exceeded 50%. The overall average number of procedures was 1.51 (95% CI 1.36 to 1.67). In PAF patients, the average number of procedures was 1.45 (95% CI 1.31 to 1.59) compared with 1.67 (95% CI 1.31 to 2.06) in NPAF patients (*P*=0.2).

### Late Recurrence After AF Ablation

**Figure 4.**
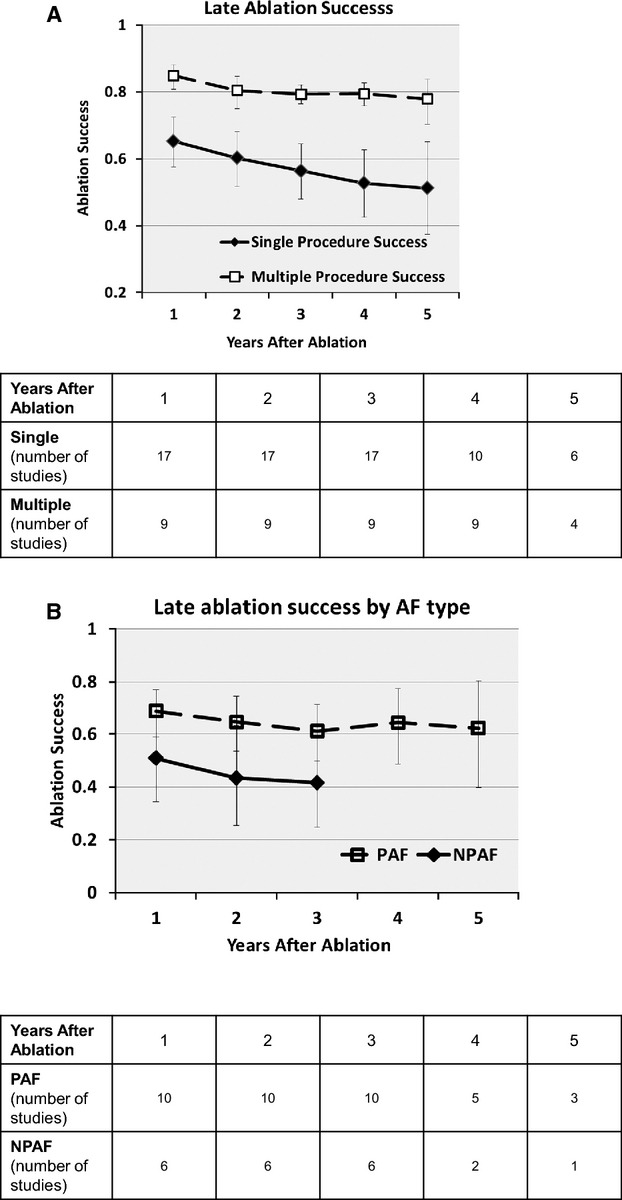
A, Annualized single‐ and multiple‐ procedure arrhythmia‐free success were calculated (subtable number of studies at each year after ablation). B, Annualized PAF and NPAF single‐procedure arrhythmia‐free success were calculated (subtable number of studies at each year after ablation). Meta‐analysis for NPAF was not performed beyond 4 years, because only 2 studies reported at this duration of follow‐up. PAF indicates paroxysmal atrial fibrillation; NPAF, nonparoxysmal atrial fibrillation.

To evaluate the timing of late recurrence, pooled estimates of single‐ and multiple‐procedure arrhythmia‐free success were evaluated for the subset of studies providing yearly follow‐up data at up to 5 years from index ablation (Figure 4). After a single procedure, the 1‐year success rate in these studies was 65.3% (95% CI 57.5% to 72.4%), which decreased to 56.4% (95% CI 47.9% to 64.5%) at 3 years and stabilized at 51.2% (95% CI 37.3% to 65.0%) at 5 years (Figure 4A). For multiple‐procedure success, the 1‐year success rate was 85.7% (95% CI 81.9% to 88.7%), which decreased to 79.3% (95% CI 76.3% to 82.0%) at 3 years and 77.8% (95% CI 70.3% to 83.8%) at 5 years (Figure 4A).

When grouped by AF type, single‐procedure success data were available from 16 studies for 1 to 3 years, 7 studies at 4 years, and 4 studies at 5 years. For PAF, single‐procedure success was 68.6% (95% CI 58.9% to 77.0%) at 1 year, 61.1% (95% CI 49.8% to 71.2%) at 3 years, and 62.3% (95% CI 39.8% to 80.5%) at 5 years (Figure 4B). For NPAF, single‐procedure success was 50.8% (95% CI 34.3% to 67.2%) at 1 year and 41.6% (95% CI 24.7% to 60.8%) at 3 years. Meta‐analysis was not performed after 3 years because <3 studies were present beyond this time point.

### Predictors of Recurrent Arrhythmia

We evaluated predictors of success using meta‐regression to explore study‐level covariates responsible for between‐study heterogeneity. We modeled the late single‐procedure success and multiple‐procedure success considering mean age, left atrial size, sex, and proportion of PAF as study‐level covariates. None of the study‐level covariates was predictive of single‐procedure or multiple‐procedure success. Thirteen individual studies reported predictors of recurrence in AF ablation in univariate and/or multivariate analysis (Table 3). Commonly identified variables predictive of AF recurrence included NPAF, left ventricular systolic dysfunction or heart failure, structural or valvular heart disease, and duration of AF.

**Table 3.** Risk Factors for Recurrence or Success After AF Ablation Were Presented for 13 Studies

Study	Predictive Model	Covariates Predictive of Reurrence/Success
Pappone, 2003^9^	Cox proportional hazards	LA diameter >45 mm predicted recurrence
Pratola, 2008^9^	Cochran‐Mantel‐Haenzel statistic	Age, presence of recurrent AF in 2 to 6 mo after ablation predicted recurrence
Sartini, 2008^9^	Cox proportional hazards	Age, time of AF, number of drugs and associated flutter, delivery power predicted recurrence
Shah, 2008^9^	Cox proportional hazards	Hypertension, hyperlipidemia predicted recurrence
Sawhney, 2009^9^	Cox proportional hazards	Hypertension predicted recurrence
Bhargava, 2009^9^	Cox proportional hazards	NPAF predicted recurrence
Bertgalia, 2010^9^	Cox proportional hazards	No variables identified predictive of recurrence
Tzou, 2010^9^	Cox proportional hazards	PAF, smaller LA size, fewer AF triggers, fewer PVs isolated predicted success
Hunter, 2010^9^	Cox proportional hazards	Structural heart disease, persistent AF, and female sex predicted recurrence
Weerasooriya, 2011^9^	Cox proportional hazards	Long‐standing persistent AF, valvular heart disease, nonischemic dilated cardiomyopathy predicted recurrence
Hussein, 2011^9^	Cox proportional hazards	Male, older age, higher BMI, NPAF, hypertension, lower LVEF, hsCRP, BNP predictive of early recurrenceAge, NPAF, left atrial size predicted late recurrence
Rostock, 2011^9^	Cox proportional hazards	Male sex, duration of persistent AF >6 mo, congestive heart failure, shorter AFCL predicted recurrenceAF termination predicted success
Winkle, 2011^9^	Cox proportional hazards	Age, left atrial size, female sex, long‐standing persistent AF, persistent AF, presence of CAD, predicted recurrence

AF indicates atrial fibrillation; LA, left atrial; NPAF, nonparoxysmal AF; PAF, paroxysmal AF; PV, pulmonary vein; BMI, body mass index; LVEF, left ventricular ejection fraction; hsCRP, high sensitivity C‐reactive protein; BNP, B‐type natriuretic peptide; AFCL, atrial fibrillation cycle length; CAD, coronary artery disease.

### Mechanisms of Recurrence

Mechanisms of recurrence were reported in 5 studies.^16–17,19,21–22^ In these 5 studies, 3 of which reported data exclusively from PAF patients, PV reconnection was noted in 417 of 423 patients who underwent repeat ablation; the rate of ≥1 PV reconnection was 97.2% (95% CI 92.7% to 99.0%). Insufficient data were available in these studies to report on the proportion of PVs undergoing reconnection or to permit stratification of reconnection rates based on type of AF or ablation strategy or technology.

### Periprocedural Complications

Periprocedural complications were reported heterogeneously across studies. Reported complications are shown in Table 4. Serious complications noted in the studies included cerebrovascular accident, PV stenosis, atrioesophageal fistula, and cardiac tamponade. Overall rates of serious complications appeared to be low (Table 4).

**Table 4.** Complications of Catheter Ablation in the Included Studies

Study	N	Complications
Pappone, 2003^9^	589	Not reported
Pratola, 2008^9^	72	1 hematoma, 1 cardiac tamponade, 1 acute myocardial infarction
Sartini, 2008^9^	139	1 transient ischemic attack, 1 acute myocardial infarction, 1 atrioesophageal fistula causing death, 5 cardiac tamphonade, 1 deep venous thrombosis
Shah, 2008^9^	264	Not reported
Katritisis, 2008^9^	39	1 cardiac tamponade
Fiala, 2008^9^	110	1 pseudoaneurysm, 1 stroke
Gaita, 2008^9^	204	2 transient ischemic attacks, 1 pseudoaneurysm, 1 esophageal ulceration
Sawhney, 2009^9^	71	1 femoral hematoma, 2 pseudoaneurysms
Bhargava, 2009^9^	1404	5 cardiac tamponades, 6 cerebrovascular events, 18 pulmonary vein stenoses, 1 hemorrhagic stroke
Bertgalia, 2010^9^	177	Not reported
Tzou, 2010^9^	123	Not reported
Hunter, 2010^9^	285	3 cerebrovascular events, 9 cardiac tamponades, 3 pulmonary vein stenoses, 77 groin hematomas, 1 pseudoaneurysm
Ouyang, 2010^9^	177	1 noninfectious pericarditis, 1 asymptomatic pulmonary vein stenosis
Medi, 2011^9^	100	No complications
Matsuo, 2011^9^	260	2 cerebrovascular events, 2 cardiac tamponades, 1 pseudoaneurysm
Weerasooriya, 2011^9^	100	3 cardiac tamponades, 3 pericardial effusions, 1 asymptomatic pulmonary vein stenosis, 1 pseudoaneurysm, 1 anaphylaxis, 1 ventricular fibrillation secondary to direct current cardioversion
Hussein, 2011^9^	831	1 arteriovenous fistula, 1 cardiac tamponade, 3 cerebrovascular events, 3 groin hematomas, 6 asymptomatic pulmonary vein stenoses
Rostock, 2011^9^	395	3 left atrial appendage isolations, 6 pacemaker implants due to sinus arrest, 4 cardiac tamponades, 1 transient ischaemic attack
Winkle, 2011^9^	893	3 strokes, 1 pulmonary vein stenosis; other complications not specified

### Publication Bias

To evaluate the included studies for publication bias, we constructed funnel plots for 12‐month success, late success, and multiple‐procedure late success (Appendix). There was some suggestion of an association between the log odds of success and the standard error of the log odds, particularly for late success, with larger, more precise studies tending to report higher success rates.

## Discussion

In this systematic review, we found that AF ablation may lead to long‐term freedom from atrial arrhythmia that is maintained at follow‐up of ≥3 years. The principal findings were that (1) a single ablation procedure may be sufficient to achieve freedom from atrial arrhythmia in ≈50% of patients, although substantial heterogeneity was noted (I^2^≥50%); (2) multiple procedures will be required to achieve control of AF in many patients, but ≈80% of patients will achieve long‐term freedom from atrial arrhythmia; and (3) although there is an incidence of late recurrence in initially successfully ablated patients, there is relative stability of arrhythmia‐free survival at late‐term follow‐up of 5 years.

### Long‐term Ablation Efficacy

Until very recently, few data have been available on AF ablation outcomes beyond 3 years after the index procedure. Indeed, current guidelines define “very late” recurrence as atrial arrhythmia >1 year after ablation and recommend follow‐up until 2 years.^2^ In our study, we addressed the issue of AF ablation outcomes at ≥3 years of follow‐up. We used drug‐free success data, where available, and follow‐up intensity was determined by individual study design. A particular consideration in a study such as this one is that the studies were reports generated at highly experienced referral centers with considerable experience in the application of AF abation. Ablation procedures were performed by experienced operators in selected AF patients. An interesting observation in our study was that the funnel plots of procedure outcomes in larger studies tended to have higher rates of success, perhaps reflecting an experience effect. However, an alternative interpretation raised by these data is that of ascertainment bias, with the possibility that different results would be achieved for procedures undertaken in lower‐volume, less‐experienced clinical centers.

Single‐procedure ablation success was achieved in ≈50% of patients, although, importantly, there was significant heterogeneity in single‐procedure outcomes in the included studies. With the inclusion of multiple procedures, ≈80% of patients achieved long‐term freedom from atrial arrhythmia.

To evaluate the long‐term stability of AF ablation success, we evaluated the annualized arrhythmia‐free success of AF ablation from 1 to 5 years. Both single‐ and multiple‐procedure success rates showed relative stability at >3 years after index ablation. Including multiple procedures, ≈80% of patients in the included studies were free of atrial arrhythmia at long‐term follow‐up. These data combined suggest that medium‐term ablation success appears to portend relative stability of long‐term efficacy of AF ablation but with a significant residual risk of recurrence affecting a significant minority of patients.

### Impact of Type of AF

The results of our study confirm previous data on the importance of AF classification to outcomes after AF ablation. The PAF ablation cohort had superior single‐procedure success compared with NPAF patients. A minority of NPAF patients in the current study achieved rhythm control with a single procedure, with long‐term rhythm control typically requiring multiple procedures. In our study, NPAF single‐procedure outcomes were surprisingly statistically not different compared with PAF outcomes. It appears that this unexpected finding is related to the substantial between‐study heterogeneity of procedural outcomes in both PAF and NPAF cohorts, as NPAF outcomes were significantly worse than those for PAF patients in all studies reporting success data for both types of patients. It should also be emphasized that long‐term outcome data for NPAF patients that were available were derived from a subset of included studies. Importantly, ≈80% of PAF and NPAF patients achieved durable sinus rhythm control at long‐term follow‐up, with the inclusion of multiple procedures. Few studies reported long‐term outcomes beyond 3 years in NPAF patients, suggesting that further data may be required to definitively assess the long‐term efficacy of ablation in this group.

### Mechanisms of Recurrence

The mechanism of recurrence in included studies was overwhelmingly related to PV reconnection, derived from the subset of studies reporting procedural data on this outcome. High rates of PV reconnection appear to occur in both PAF and NPAF patients. This would suggest that improvement in overall outcomes of the procedure may require improvements in both technique and technology. The extent of data available did not allow identification of other factors that may contribute to recurrence in these studies. Nevertheless, a number of studies have demonstrated the importance of patient substrate factors including type of AF, left atrial diameter, structural heart disease, left ventricular dysfunction, hypertension, obesity, and obstructive sleep apnea.^28–31^

### Clinical Parameters Related to Between‐Study Heterogeneity

In the current study, substantial heterogeneity was observed in single‐procedure outcome (Figure 2), with a significant risk of late recurrence after index ablation (Figure 4A). The wide disparity in reported success rates between the included studies is in itself an outcome of significant importance. We explored possible reasons for recurrence with a meta‐regression analysis using age, sex, percentage of PAF patients, left atrial diameter, and mean left ventricular ejection fraction as moderator variables. None of the study variables was found to be statistically predictive of short‐ or late‐term success outcomes after AF ablation. At the study level, a wide array of covariates were found to be associated with ablation outcomes (Table 3), suggesting that further information is required to precisely predict factors related to prognosis in individual patients.

Although we were not able to provide a statistical explanation for between‐study heterogeneity, we suggest that several areas of clinical difference between studies may account for differences in outcomes: (1) differences in patient population receiving ablation, (2) differences in technique and technology used during ablation, (3) differences in the use and reporting of antiarrhythmic drug therapy after ablation, (4) follow‐up frequency and intensity, (5) definitions of procedural success or failure, and (6) differences in the availability and timing of repeat procedures. Standardization of reporting of these clinical parameters is an area that may need to be addressed in future revisions to current clinical guidelines. An area that may require particular emphasis is the need for long‐term follow‐up of AF ablation patients, which we believe may need to be extended beyond the 12‐month window postprocedure recommended in current clinical guidelines.^2^

### Comparisons to Previous Meta‐analyses

Over the years, a number of studies have addressed the shorter‐term outcomes,^32–34^ predictors of recurrence,^28–29^ or impact of specific types of ablation strategy.^35–36^ The current study, for the first time, addresses the significant clinical issue of the long‐term clinical outcomes of AF ablation, which has only recently become possible due to the availability of long‐term clinical follow‐up data after AF ablation.

### Clinical Implications

The long‐term results of AF ablation are critically important not only for individual patient prognosis and clinical decision making but also for determining the role of reimbursement policy for the procedure. The data presented in the current study suggest that long‐term freedom of atrial arrhythmia can be achieved in the majority of AF cases, taking into account the need for multiple procedures in a significant proportion of patients.

### Study Limitations

The results of this report were compiled using meta‐analyses of primarily nonrandomized observational data, rather than randomized controlled trial data, with significant limitations in study quality, thereby having some risk of bias.^5,37^ The technique of meta‐analysis was originally developed for prospectively conducted randomized controlled trials, which represent the highest quality of evidence evaluating the efficacy of clinical interventions. In recent years, however, meta‐analysis has become accepted in the literature to aggregate results from observational data, to facilitate synthesis of available evidence and generation of new hypotheses.^37–38^ In the case of AF ablation, the procedure is relatively new, with significant ongoing innovation in technology and technique, necessitating the inclusion of case series as well as randomized controlled trial data. In addition, the limitation of included data to published studies may lead to a risk of publication or “file drawer” bias, which may favor the publication of studies showing an improvement in outcomes.^39^ Significant heterogeneity was identified, although lack of similarity in reporting of outcome and moderator variables limited the opportunity for more detailed subgroup analyses. However, we specifically acknowledge the limitations and inherent bias that may occur with this approach. A further limitation of our study is that periprocedural complications, a critical consideration in evaluating the risks and benefits of the procedure, were variably reported in terms of level of detail, and in some studies not reported at all. The conclusions of our study are therefore predicated on explicit acknowledgment of these possible limitations in the study design.

## Conclusions

The data presented in this review showed encouraging rates of success at long‐term follow‐up after early experience with catheter ablation of AF. Although single‐procedure outcomes were associated with significant heterogeneity, with the inclusion of multiple procedures, long‐term freedom from atrial arrhythmia was achievable in the majority of patients.
